# Comparison of single– and five–fraction schedules of stereotactic body radiation therapy for central lung tumours: a single institution experience

**DOI:** 10.1017/S1460396917000061

**Published:** 2017-05-08

**Authors:** Sung Jun Ma, Yusef A. Syed, Charlotte I. Rivers, Jorge A. Gomez Suescun, Anurag K. Singh

**Affiliations:** 1 Jacob School of Medicine and Biomedical Sciences, University at Buffalo, Buffalo, NY, USA; 2 Department of Radiation Medicine, Roswell Park Cancer Institute, Buffalo, NY, USA

**Keywords:** central lung tumour, late toxicity, single fraction, stereotactic body radiation therapy

## Abstract

**Background:**

Stereotactic body radiation therapy (SBRT) is a treatment option for patients with early-stage non-small cell lung cancer who are medically inoperable or decline surgery. Here we compare the outcome of patients with centrally located lung tumours who underwent either single fraction (SF)- or five-fraction (FF-) SBRT at a single institution over 5 years.

**Methods:**

Between January 2009 and October 2014, patients with centrally located lung tumours who underwent SBRT were included in this study. Data were retrospectively collected using an institutional review board-approved database. For analysis, the Kaplan–Meier method and competing risks method were used.

**Results:**

In total, 11 patients received 26–30 Gy in 1 fraction, whereas 31 patients received 50–60 Gy (median 55 Gy) in 5 fractions. After a median follow-up of 12 months for SF-SBRT and 17 months for FF-SBRT groups (*p* = 0.64), 1-year overall survival rates were 82 and 87%, respectively. SF- and FF-SBRT groups showed no significant difference in grade 3+ toxicity (*p* = 0·28). The only grade 4 toxicity (*n* = 1) was reported in the SF-SBRT group. All toxicities occurred >12 months after the SBRT.

**Conclusions:**

SF- and FF-SBRT have comparable overall survival. SF-SBRT may have some utility for patients unable to have multi-fraction SBRT.

## BACKGROUND

For early-stage non-small cell lung cancer (NSCLC), surgery remains the standard of care with a 5-year survival rate of 46–63%.^1^ However, many patients are not surgical candidates due to significant medical comorbidities. Stereotactic body radiation therapy (SBRT) is a non-invasive treatment approach that offers an alternative to surgery in medically inoperable patients. SBRT has been shown to provide local control (LC) rates of 90%, with survival rates comparable with those with surgery alone.^2–5^

The toxicity of SBRT may differ based on tumour location relative to the proximal bronchial tree.^6^ Previous studies have shown that for peripherally located tumours (>2 cm from the proximal bronchial tree), single-fraction (SF) SBRT is well tolerated with relatively low toxicity.^7–11^ Centrally located tumours (<2 cm from the proximal bronchial tree) treated with multiple-fraction SBRT are perhaps similarly well tolerated compared to peripheral lung tumours,^12^ whereas SF-SBRT for central lung tumours have been evaluated in relatively fewer studies.^13,14^

Over a 5-year period at Roswell Park Cancer Institute (RPCI), 42 patients received either SF or five-fraction (FF-) SBRT for centrally located lung tumours. In this retrospective review, we evaluate the clinical outcome of these patients, including survival rates and toxicities.

## METHODS

### Patients

A retrospective chart review was conducted on patients who underwent definitive treatment using SF- and FF-SBRT for central lung tumours at RPCI between January 2009 and October 2014. All information was collected and maintained in a database approved by the institutional review board at RPCI.

Before SBRT treatment, all patients underwent initial consultation for complete history and physical examination. Imaging included computed tomography (CT) and/or fluorodeoxyglucose positron emission tomography (FDG-PET) in most cases.

### Treatment planning

CT simulation for all patients was performed with patients in the supine position, using a Body Fix (Elekta, Stockholm, Sweden) immobiliser. Respiratory motion was assessed using Real-Time Position Management by Varian Medical System (Palo Alto, CA, USA). All patients underwent 4D CT to evaluate tumour movement with respiration and to generate an internal target volume (ITV).

Treatment planning was per RTOG 0915 for SF-SBRT and per RTOG 0813 for FF-SBRT,^9,10,15^ using a 3D-conformal approach with 11 non-coplanar fields on all patients, except for five patients in the FF-SBRT group who underwent volumetric-modulated arc therapy. No intensity-modulated radiation therapy was used. Gross tumour volume (GTV) was identified and outlined on each CT slice by an appropriately trained physician. Tumours were identified using lung windows, as well as soft tissue windows as necessary to distinguish tumour from adjacent atelectasis or vessels. The clinical target volume was defined as equal to GTV. An ITV surrounding the GTV was generated as described above using 4D CT to account for tumour motion. The planning target volume (PTV) was created by adding a uniform 0·5 cm margin to the ITV.

Of 11 patients who received SF-SBRT, 10 patients received 30 Gy (without heterogeneity corrections), and the remaining one patient received 26 Gy (with heterogeneity corrections). All FF-SBRT patients were planned without heterogeneity correction. Dose was prescribed to the prescription line at the edge of the PTV. Dose conformality and normal tissue constraints were as per RTOG 0915 for SF-SBRT and per RTOG 0813 for FF-SBRT. The prescription isodose surface was required to be greater than or equal to 60%, and <90% of the maximum dose. In all, 99% of the PTV was required to receive ≥90% of the prescription dose.

### Follow-up

Patients were scheduled for follow-up at 1·5, 3, and 6 months post-treatment. Subsequent follow-ups were performed every 6 months for 2 years. Tumour response to treatment was assessed with a PET/CT at the 1-year follow-up and surveillance CT imaging of the chest at all other follow-up visits.

### Outcome assessment

Local failure was defined as recurrence within the involved lobe or PTV. Nodal failure was defined as recurrence in regional lymph nodes along the natural lymphatic drainage based on the location of primary tumour. Distant failure was defined as recurrence in uninvolved lobes or other organs, including new sites of distant failure for patients with oligometastasis in lungs.

Toxicity was analysed retrospectively from the clinical database using the Common Terminology Criteria for Adverse Events version 4·0. Patients with multiple toxicities were counted once using the highest toxicity grade only. All severe adverse events following treatment were reported regardless of presumed aetiology. Only toxicities directly attributable to radiation therapy were included for analysis. Other adverse events that were deemed to be caused by preceding respiratory comorbidities (e.g., pneumonia, chronic obstructive pulmonary disease (COPD) exacerbation and other respiratory infections) were excluded from this analysis.

### Statistical methods

Overall survival (OS) and progression-free survival (PFS) were assessed using the Kaplan–Meier method, and treatment groups were compared using log-rank tests. Local failure, nodal failure and distant failure were calculated using the competing risks method, and treatment groups were compared using Gray’s tests. Differences in grade 3+ toxicity were calculated using Fisher’s exact test. Differences between treatment groups were evaluated with Fisher’s exact test for categorical variables and Mann–Whitney *U* test for continuous variables. All *p* values were two-sided, and *p* values <0·05 were considered significant. R software version 3·3·1 (R Foundation for Statistical Computing, Vienna, Austria) was used for statistical analysis.

## RESULTS

### Baseline characteristics

A total of 42 patients with central lung tumours, including NSCLC (*n* = 35), limited-stage small cell lung cancer (SCLC, *n* = 1), or lung oligometastases (*n* = 6) were evaluated. Patient characteristics are described in Table 1. The median age was 75·5 (range 50–89), median Karnofsky Performance Status was 80 (range 60–100), and the median smoking in pack-year was 50 (range 3–150). The median tumour diameter was 2·3 cm (range 0·1–4). The majority of lung tumours were adenocarcinoma and squamous cell carcinoma, stage I, and clinical T1N0. Five patients in SF- and 19 patients in FF-SBRT regimen groups had no history of chemotherapy, surgery or radiation treatment affecting their lungs, whereas all other patients had previous cancer treatments, including prior lung resections and systemic chemotherapy for cancer in other organs.

In the SF-SBRT treatment group, 11 patients received 26–30 Gy (median 30 Gy). In the FF-SBRT group, 31 patients received 50–60 Gy (median 55 Gy). Two patients in the SF-SBRT group and five patients in the FF-SBRT group declined surgery, whereas the remaining patients were medically inoperable due to poor performance status and limited pulmonary or cardiac function.

Malignancy was confirmed with biopsy in all but one SF-SBRT patient, for whom the lesion was determined to be malignant by PET/CT scan showing a new 1-cm lesion with a standardised uptake value (SUV) of 3·4.

### Outcomes

**Figure 1.**
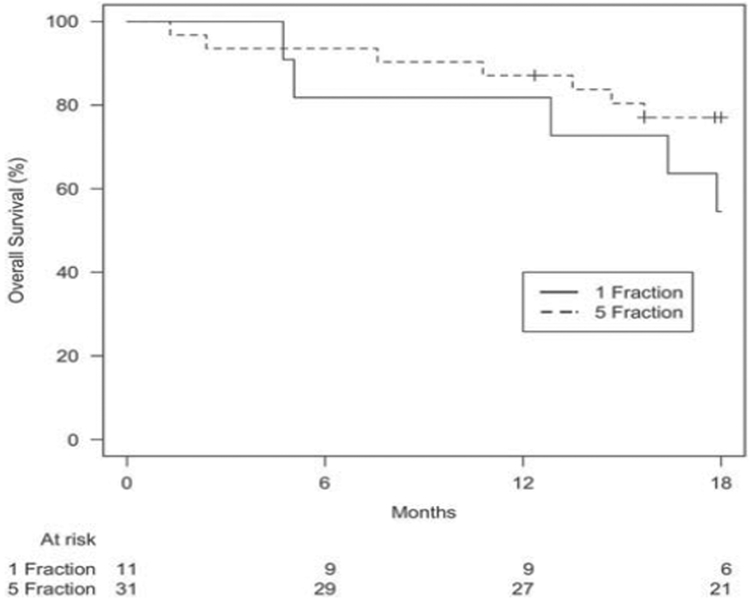
*Overall survival based on treatment group.* p *Value: 0·061.*

**Figure 2.**
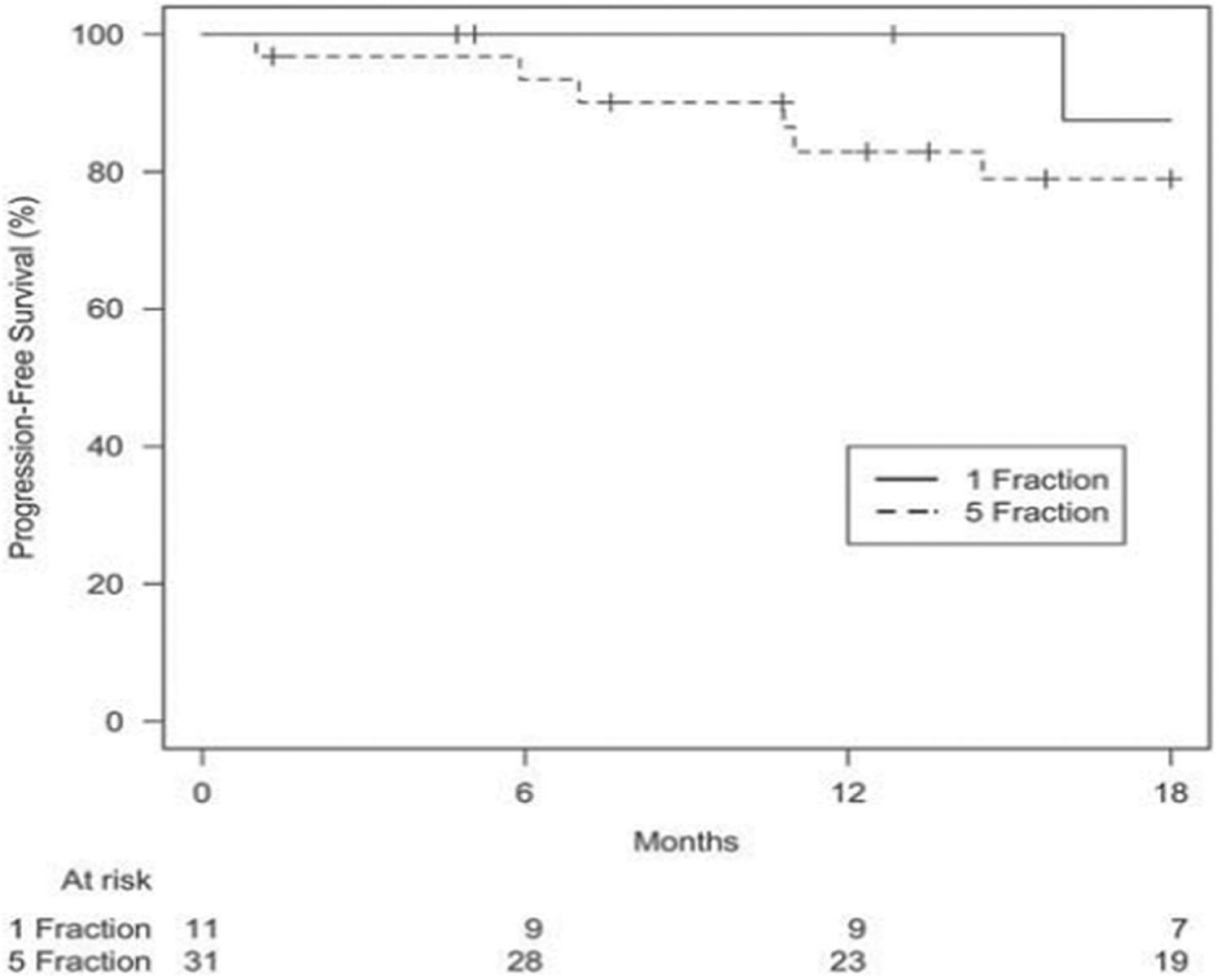
*Progression-free survival based on treatment group.* p *Value: 0·47.*

**Figure 3.**
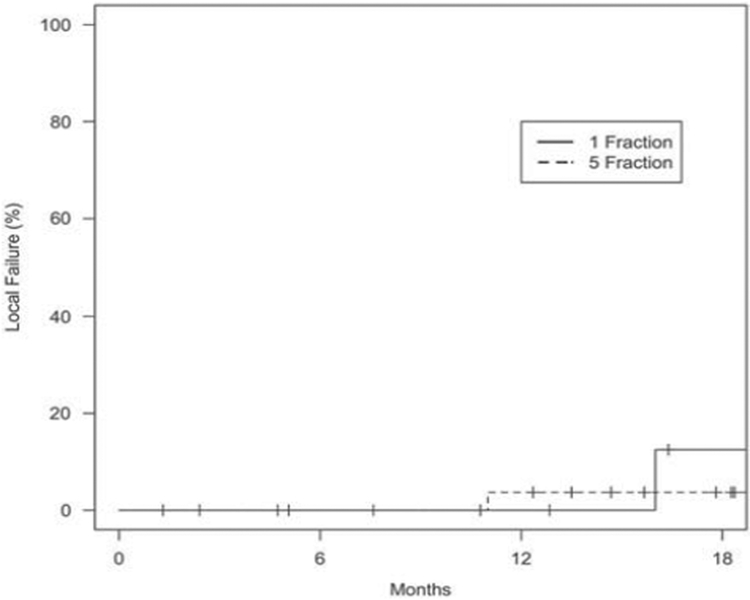
*Local failure based on treatment group.* p *Value: 0·43.*

**Figure 4.**
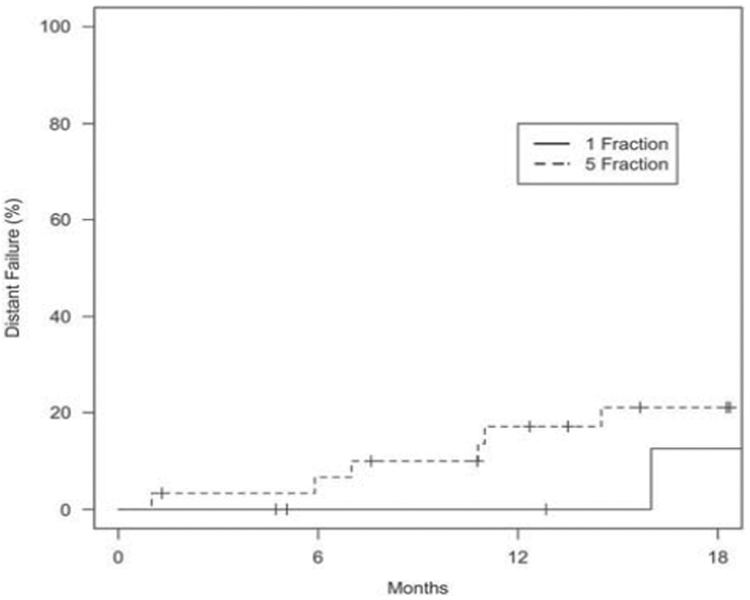
*Distant only failure based on treatment group.* p *Value: 0·45.*

After a median follow-up of 12 months (range 1·4–41·2) for the SF-SBRT and 17 months (range 0–60·7) for the FF-SBRT groups (*p* = 0·64), median OS was 27 and 25 months, respectively. One-year OS rates for the SF- and FF-SBRT groups were 82 and 87%, respectively. The 1-year LC rate was 100% for the SF-SBRT group, and 96% for FF-SBRT group. There was no statistically significant difference in OS (*p* = 0·061), PFS (*p* = 0·47), local failure (*p* = 0·43), nodal failure (*p* = 0·42), and distant failure (*p* = 0·45) at 18 months (Figures 1–4).

### Toxicity

Two patients from each treatment group exhibited multiple grade 3–4 toxicities. These patients were counted once using the highest grade of toxicity only. Five out of 11 patients in the SF-SBRT group experienced grade 3–4 toxicities, such as bronchopulmonary haemorrhage,^16^ left vocal cord palsy^17^ and pneumonia. Eight out of 32 patients in the FF-SBRT group reported grade 3 toxicities only, including pneumonia, COPD exacerbation, pleural effusion, pulmonary embolism and non-specified respiratory infection.

Lesser toxicities considered to be caused by respiratory comorbidities were excluded from this analysis. After the exclusion of such toxicities, two out of 11 patients in the SF-SBRT group and two out of 32 patients in the FF-SBRT group experienced grade 3–4 toxicities. Characteristics of these four cases are described in Table 2. No grade 4–5 toxicities were observed in the FF-SBRT group. The only grade 4 toxicity (*n* = 1) occurred in SF-SBRT group, whereas no grade 5 toxicity was observed. All toxicities resolved, except for one case of grade 4 bronchopulmonary haemorrhage necessitating pneumonectomy, which resulted in surgical complications leading ultimately to death.^16^ SF- and FF-SBRT groups showed no significant difference in grade 3+ toxicity (*n* = 2 for SF-SBRT, *n* = 2 for FF-SBRT, *p* = 0·28). Median time to radiation-induced toxicity was 14·6 months and all toxicities occurred at least 12 months after SBRT.

## DISCUSSION

This retrospective review of 42 patients with central lung tumours compares clinical outcomes for those treated with SF-SBRT versus those treated with FF-SBRT. To our knowledge, this is the first such comparison that has been reported in the literature.

Our SF- and FF-SBRT groups showed no significant difference in OS and LC. The 1-year OS and LC rate in our SF-SBRT cohort was comparable with those reported elsewhere.^13,14^ Similarly, OS and LC rates in our FF-SBRT cohort were consistent with past studies.^18,19^

In our patients, SF- and FF-SBRT had no statistically significant difference in the prevalence of grade 3+ toxicity. However, a single case of grade 4 toxicity was reported in the SF-SBRT group, whereas grade 3 toxicities only were reported in the FF-SBRT group. Other multi-fraction regimens, showed low overall toxicity,^18–21^ however, there area few cases of high toxicity.^22–24^ The median time to grade 3 or higher toxicity in our study was 14·6 months from treatment, which is consistent with other studies.^6,22,24^ When treating patients with central lung tumours, some degree of mortality risk seems to be inevitable regardless of whether SBRT^25^ or pneumonectomy^26^ is performed.

In addition to the inherent limitations of any retrospective review, our patient cohort was very small. Although not statistically significant, both cohorts had heterogeneous tumour types (including early-stage NSCLC and oligometastases) and different length of follow-up.

Several prospective, multicentric phase I–II studies are in progress to further examine multi-fraction SBRT for central lung tumours, including RTOG 0813^15^ and European Organization for Research and Treatment of Cancer 22113–08113.^27^ These studies may further elucidate OS, patterns of failure and toxicities seen in the treatment of central lung tumours.

## CONCLUSION

Central SF- and FF-SBRT had comparable OS and LC. Though not currently being studied in prospective trials, SF-SBRT may have some utility for patients unable to have multi-fraction SBRT.

## Figures and Tables

**Table 1.** Baseline characteristics of patients and tumours

Baseline characteristics	26–30 Gy in 1 fx (*n*=11) number (%)	50–60 Gy in 5 fx (*n*=31) number (%)	*p* value
Follow-up (months)			
Median	12	17	NS (0·64)
Range	1·4–41·2	0–60·7	
Age			
Median	78	74	NS (0·32)
Range	56–84	50–89	
Gender			
Male	9 (82)	14 (45)	NS (0·08)
Female	2 (18)	17 (55)	
KPS			
Median	80	80	NS (0·37)
Range	60–90	70–100	
Smoking (pack-year)			
Median	68·75	50	NS (0·12)
Reason for SBRT			
Declined surgery	2 (18)	5(16)	NS (1)
Non-surgical candidate	9 (82)	26 (84)	
Type of tumour			
Primary	8(73)	28 (90)	NS (0·31)
Oligometastasis	3(27)	3 (10)	
Tumour size (cm)			
Median	2·6	2·1	NS (0·50)
Range	0·1–3·9	0·7–4	
AJCC stage group			
NA	4(36)	3(10)	NS (0·20)
1A-B	6 (55)	28 (90)	
3B	1 (9)	0 (0)	
Tumor Stage			
NA	4 (36)	3(10)	NS (0·65)
1A-B	6 (55)	20 (64)	
2A-B	1 (9)	8 (26)	
Nodal Stage			
NA	4 (36)	3 (10)	NS (0·20)
0	6 (55)	28 (90)	
3	1 (9)	0 (0)	
Lung treatment history			
None	5 (45)	19 (61)	NS (0·48)
Prior chemotherapy	4 (36)	4(13)	(any treatment history vs. none)
Prior lung surgery	2 (18)	9 (29)	
Prior lung radiation	1 (9)	2(6)	

*Abbreviations*: Fx, fraction; NS, not significant; KPS, Karnofsky performance status; NA, not available or not specified; SBRT, stereotactic body radiation therapy; AJCC, American Joint Committee on Cancer.

**Table 2.** Characteristics of patients with grade 3+ toxicity

No.	Gender	Tumour type	Location	Tumour size (cm)	Total dose/fx (Gy/Fx)	History	Follow-up (months)	Toxicity grade	Toxicity	Time to toxicity (months)
1	M	Mets	LUL	2·7	30/1	SBRT 60 Gy in 3 fx (LLL)	41·2	3	Left vocal cord palsy	14·5
2	M	Mets	Right hilum	3	26/1	Chemo-radiation (70 Gy in 35 fx, cancer in the base of tongue)	12·8	4	Bronchopulmonary haemorrhage	12·6
3	F	NSCLC	LUL	1·6	52·5/5	Surgery (RUL)	33·7	3	Pulmonary embolism	19·1
4	M	NSCLC	RML	2·3	55/5	Surgery (LUL)	22·9	3	Pleural effusion	14·6

*Abbreviations*: Fx, fraction; M, male; F, female; mets, pulmonary oligometastasis; SBRT, stereotactic body radiation therapy; NSCLC, primary non-small cell lung cancer; LUL, left upper lobe; RML, right middle lobe; LLL, left lower lobe; RUL, right upper lobe.
